# Editorial: On the frontier of a plant’s dilemma: Exploring the molecular basis ofgrowth versus defense antagonism 

**DOI:** 10.3389/fpls.2022.1067441

**Published:** 2022-11-03

**Authors:** Ian Tomoharu Major, Marcelo Lattarulo Campos

**Affiliations:** ^1^ Laurentian Forestry Centre, Natural Resources Canada, Québec, QC, Canada; ^2^ Integrative Plant Research Laboratory, Departamento de Botânica e Ecologia, Instituto de Biociências, Universidade Federal de Mato Grosso, Cuiabá, Brazil

**Keywords:** growth-defense tradeoffs, plant hormones, signaling, plant stress responses, plant defense/immunity

Pest and pathogen attacks are responsible for major agricultural losses and are critical obstacles to global food security, a problem that is expected to be exacerbated with changing climates and increasing populations (Deutsch et al., 2018; Savary et al., 2019; Delgado-Baquerizo et al., 2020; Jones, 2021; Ristaino et al., 2021). A major hurdle to increasing plant resilience against biotic attackers is the antagonism between growth and defense, which is a well-documented physiological bottleneck where the activation of growth processes has detrimental effects on defenses or vice-versa (Smedegaard-Petersen and Tolstrup, 1985; Herms and Mattson, 1992; Huot et al., 2014; Sestari & Campos, 2022). Besides its agronomic relevance, this “dilemma of plants” also has profound impacts on natural ecosystems, where it serves as a major factor defining the composition of plants and their enemies (Zust et al., 2012; Cappelli et al., 2020). Despite the ecological and economic relevance of plant growth-defense balance, the molecular mechanisms controlling their antagonism are just now being uncovered. This Research Topic of Frontiers in Plant Science presents recent findings on the molecular basis of this dilemma. Subjects extend from the identification of novel genes and molecules that modulate growth and defense to the molecular mechanisms associated with plant responses against viral infections.

The identification of genetic players that regulate both growth and defense simultaneously is important for understanding, and perhaps eventually manipulating, the balance of these antagonistic processes (Karasov et al., 2017; Monson et al., 2022). In this Research Topic, Khan et al. demonstrates that ILL6, an aminohydrolase involved with turnover of the defense-related hormone jasmonate (JA; Widemann et al., 2013), may also modulate growth and disease resistance in the plant model *Arabidopsis thaliana*. The authors characterized a loss of function *atill6* mutant, which showed longer shoots compared to wild-type (WT) plants, as well as longer roots under oxidative-stress conditions. Consistent with growth-defense antagonism, *atill6* mutants were more susceptible to infection by the bacterial pathogen *Pseudomonas syringae* DC3000, supporting increased bacterial growth and reduced expression of defense marker genes compared to WT plants. Taken together, these data suggest that *ATILL6* modulates growth-defense balance in *A. thaliana*, acting as a positive regulator of defense responses to microbial pathogens while also influencing root and shoot growth under normal and abiotic stress conditions. Although additional work is needed to fully grasp the role of ATILL6 in plant development, it is tempting to speculate that this gene may serve as a potential target for future research aiming to uncouple growth and defense tradeoffs in *A. thaliana* and even other plant species.

Among the genetic controls already identified as key regulators of growth-defense balance, the MYC transcription factors (TFs) stand out as examples whose function is well-studied and understood. MYC2, MYC3 and MYC4 are the main TFs involved with the activation of the JA signaling pathway, thus being positively associated with upregulation of defense responses, but negative regulators of growth (Major et al., 2017; Ortigosa et al., 2020; Guo et al., 2022). In this Research Topic, Wang et al. examined several *myc* single and higher order mutants in *A. thaliana* to examine the individual roles of these TFs and found that, consistent with previous work, MYC3 had the strongest effect on insect resistance (Fernández-Calvo et al., 2011). To identify genes regulated by each MYC, the authors then profiled the transcriptome of *myc* double mutants, such that each mutant has a single WT MYC, and found that MYC3 also had the strongest influence on the expression of genes associated with insect resistance. Further analysis showed that genes associated with MYC3 regulation and insect resistance were enriched in flavonoid biosynthesis and some growth-related processes, particularly auxin. The authors showed that several flavonoid biosynthesis genes were co-expressed with these auxin-associated genes, supporting the notion that flavonoids may be molecular links between growth and defense (Peer and Murphy, 2007). To test this hypothesis, the authors evaluated how a flavonoid synthesis mutant, *chalcone synthase* (*chs*), responded to mechanical wounding, which is a proxy for insect attack known to activate defense responses and inhibit growth in *A. thaliana* (Zhang and Turner, 2008). The wounding treatment restricted biomass more strongly in *chs* than WT, while there was no difference in plant growth before wounding, suggesting that flavonoids may alleviate growth inhibition caused by wounding in *Arabidopsis*.

On the topic of defense signals that also influence growth, Shields et al. provide a comprehensive review of the interconnection between two central immune signals, salicylic acid (SA) and *N-*hydroxipipecolic acid (NHP), which act as a hub that regulates the interface between growth and defense in plants. The authors highlight how SA and NHP act cooperatively and synergistically to induce defense responses against microbial pathogens and describe how increased SA and NHP levels attenuate plant growth and development. The manuscript provides an updated compilation of the molecular mechanisms involved with such growth repression, highlighting genes that may be the focus of future work aimed at mitigating the growth restriction associated with plant immunity. Of particular interest is the extent to which SA and NHP crosstalk with signaling components of growth-related plant hormones (such as brassinosteroids and auxins), providing a complex overview of how growth-defense balance is controlled by a multi-layered and multi-faceted network of hormonal pathways.

Lastly, Hayano-Saito and Hayashi identified and characterized the *Stvb-i* resistance gene, which has provided sustained resistance in rice cultivars against infection by *Rice stripe virus* (RSV), the causal agent of rice stripe disease. This illness stunts plant growth, reduces panicle formation and grain filling, thus largely affecting productivity (Hayano-Saito et al., 2000). While viruses are responsible for almost half of emerging infections plant diseases, with the potential to cause agricultural and economical loss worldwide (Anderson et al., 2004; Jones, 2021), the tradeoffs between plant growth and immunity against viruses remain largely unknown. The authors found that *Stvb-i* limits growth stunting by RSV, and moreover contributes by itself to growth and development in the absence of infection, as *Stvb-i-*silenced lines are significantly stunned, with abnormal bending of the lamina joint and fewer tillers compared to WT. Surprisingly, the authors also noted that the growth difference between *Stvb-i-*silenced lines and WT was exacerbated at higher temperatures, suggesting that *Stvb-i* may also be involved with heat stress responses. Taken together, data presented by Hayano-Saito and Hayashi demonstrate that, besides providing resistance to viral infection, *Stvb-i* also has an important role in rice growth and development. It may also suggest that stabilizing plant growth could be a remarkable strategy to achieve multi-stress tolerance in plants.

The studies collected in this Research Topic of Frontiers in Plant Science present some recent findings that contribute to our growing understanding of the genes associated with the control of growth-defense balance. The potential to manipulate this trade-off offers the exciting promise that defense can be improved while minimizing growth restriction. In this sense, the identification of the genetic players involved in the antagonism of growth and defense, combined with recent advances in technologies like gene editing and omics approaches, may offer a simple strategy to eventually resolve the “plant’s dilemma” in many plant species. It is tempting to suggest that this will contribute to the next green revolution, allowing increased crop productivity while diminishing the need for inputs to manage pests and pathogens.

## Author contributions

All authors listed have made a substantial, direct, and intellectual contribution to the work and approved it for publication.

## Funding

Funding to ITM was provided by the Canadian Forest Service, Natural Resources Canada. Funding to MLC was provided by the Fundação de Amparo à Pesquisa do Estado de Mato Grosso (grant number 0209246/2021) and Conselho Nacional de Desenvolvimento Científico e Tecnológico (grant number 402160/2021-5).

## Conflict of interest

The authors declare that the research was conducted in the absence of any commercial or financial relationships that could be construed as a potential conflict of interest.

## Publisher’s note

All claims expressed in this article are solely those of the authors and do not necessarily represent those of their affiliated organizations, or those of the publisher, the editors and the reviewers. Any product that may be evaluated in this article, or claim that may be made by its manufacturer, is not guaranteed or endorsed by the publisher.
